# Usefulness of air sonographic artifacts in clinical diagnosis

**DOI:** 10.4103/mgr.MEDGASRES-D-25-00118

**Published:** 2026-01-06

**Authors:** Fikri M. Abu-Zidan, Mustafa Boraie, Arif Alper Cevik

**Affiliations:** Department of Surgery, College of Medicine and Health Sciences, United Arab Emirates University, Al-Ain, United Arab Emirates; Statistical and Research Methodology Consultant, The World Society of Emergency Surgery, Bologna, Italy; Department of Critical Care and the Intensive Care Unit, Burjeel Royal Hospital, Al-Ain, United Arab Emirates; Department of Internal Medicine, Emergency Medicine Section, College of Medicine and Health Sciences, United Arab Emirates University, Al-Ain, United Arab Emirates

In this perspective, we aim to explain the basic biophysics and clinical usefulness of gas reverberation artifacts when properly understood and utilized. This communication highlights the dynamic transferability of knowledge on the biophysics of these artifacts. A report on reverberation artifact in a horse in 1981 was transferred to human lungs after approximately two decades, which revolutionized its clinical application, and is now being transferred to the human abdomen. Three basic sonographic findings that were transferred are: (1) the enhanced pleural line with sliding lung, (2) the reverberation artifact, and (3) the lung point sign. These have the same biophysical principles as: (1) the linear hyperechogenic peritoneal stripe with the absence of peritoneal sliding, (2) the reverberation artifact, and (3) the gut point. Initial reports on the use of ultrasound to detect free intraperitoneal air were very encouraging. Nevertheless, recent studies have contradictory results. Ultrasound has been useful in detecting free intraperitoneal air in our own hands. This can be subjective depending on the operator’s experience. This highlights the need to perform large prospective multicentric cohort studies to define the role and generalizability of using ultrasound in diagnosing free intraperitoneal air.

Those who were privileged to observe the development of using Point-of-Care Ultrasound (POCUS) as a diagnostic tool in life-threatening conditions over the last four decades became convinced that it was efficient to save many lives. There was major resistance and reluctance by radiologists for its use by non-radiologists at the start. The proliferation of the utilization of POCUS both horizontally by non-radiologists in numerous diagnostic indications and vertically by refined skills in interventional procedures, like ultrasound-guided nerve blocks and insertion of central lines, was astonishing. This was possibly one of the most successful medical diagnostic and interventional developments, which improved point-of-care clinical management and outcome. Over time POCUS became a Physiological evaluation tool that gives On spot critical clinical decisions, as part of the Clinical examination which is Unique and Safe.^1^

Air was considered to be the enemy of ultrasound because it does not permit ultrasound waves to pass through, making it difficult to examine the abdomen or lungs, which contain gas. Accordingly, sonographers were searching for windows without gas to be able to visualize the intra-abdominal structures. It was only in 1995, when Professor Lichtenstein, a critical care physician from Paris, described the sonographic pattern of the lung ultrasound in humans, which is simply a reverberation sonographic artifact.^2^ Interestingly, these were previously described in a horse by Rantanen in 1981 using high-frequency probes and a B-mode ultrasound to define the lung border.^3^ Furthermore, he later recommended locating the linear ultrasound probe to be parallel to the costal margins when used, described how to diagnose a pneumothorax by ultrasound, and how to use a needle to evacuate it if no other radiological facilities were available.^4^ It is exciting to see how these skills were transferred from horses to humans almost two decades later. This has opened a new revolution in lung ultrasound, which has now become routine in every advanced critical care unit. In this perspective, we aim to explain the basic biophysics and usefulness of reverberation artifacts when transferred from the lung to the abdomen in emergency settings so that they can be understood and used by many.

**Basic ultrasound biophysics:** We have learned over time that thoroughly understanding the basic biophysics of ultrasound enforces the operator’s ability to interpret ultrasound images. Piezoelectric crystals are located inside the ultrasound probes. These are the most expensive parts of the probe. They produce high-frequency sonographic waves ranging between 2 MHz to 15 MHz and can receive the reflected ultrasound waves from the tissues, which are then processed by the ultrasound machine to black and white two-dimensional images with a thickness of 1 mm. Accordingly, this is called the brightness mode (B-mode).

**Figure 1:**
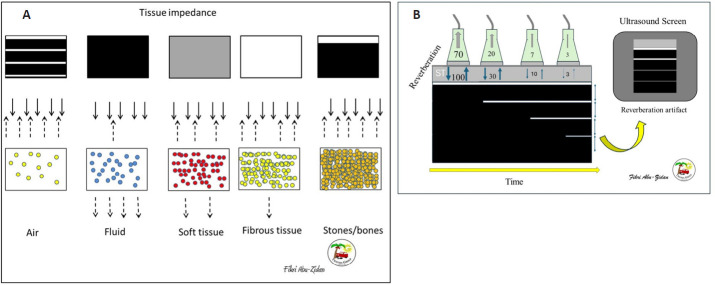
An illustration demonstrating different tissue impedances reflecting ultrasound waves and reverberation artifacts. (A) Ultrasound waves: Denser materials reflect more ultrasound waves. Accordingly, fluid (like ascites) is black (anechoic), soft tissue (like the liver) is grey, fibrous tissue (like the diaphragm) is white without a shadow, and stones are white (hyperechoic) with a shadow. Reprinted with permission from Abu-Zidan and Cevik.^1^ (B) Reverberation artifact: A theoretical example of reverberation artifact is when the probe produces 100 ultrasound waves. These waves bounce between these two interfaces while they are gradually being processed by the ultrasound machine over time. Created with Microsoft PowerPoint.

Tissue impedance will differ according to its density. The distances between particles of media will differ according to their density. As the particles are closer, the density of a media increases, with more ability to reflect ultrasound waves. To simplify the issue, the sun is very shiny while the night will be dark. The sequence of higher densities, ultrasound reflection, and black and white colors are as follows: (1) solid tissues like stones and bones are white with black shadow behind them as they do not permit the ultrasound waves to go through them, (2) fibrous tissues like the diaphragm are white (hyperechoic) without a shadow, (3) soft tissues like the liver, kidney, and spleen are grey, and (4) fluids like blood, urine, ascites, and bile are black (anechoic) (**Figure 1A**). The gas does not have a shape, and its particles have a fast movement strongly reflecting the ultrasound waves and preventing it from going through. This gives a unique image called the reverberation artifact.^1^

**The sliding lung:** There are two lung pleurae, the visceral pleura that surrounds the lung and the parietal pleura that covers the inner surface of the chest wall. Pneumothorax is the presence of air between the visceral and parietal pleurae. Since the pleura is a fibrous tissue, it will be hyperechoic, shiny, and white. When the lung expands during inspiration, the visceral pleura will move over the parietal pleura. This will appear as a real-time moving white line, which is called the “sliding sign” indicating that the visceral and parietal pleurae are touching each other. This excludes the presence of a pneumothorax.^2^ In contrast, the absence of lung sliding indicates that the lung is not moving with a differential diagnosis of (1) pneumothorax, (2) one lung intubation, (3) total lung collapse due to bronchial obstruction by a plug, or (4) adhesions between the lung and chest wall. The “lung point” sign, which is diagnostic for pneumothorax, is the point between the edge of the pneumothorax and a normal pleura, where both the non-moving and moving pleura are visualized in the same B-mode ultrasound image.

**Figure 2:**
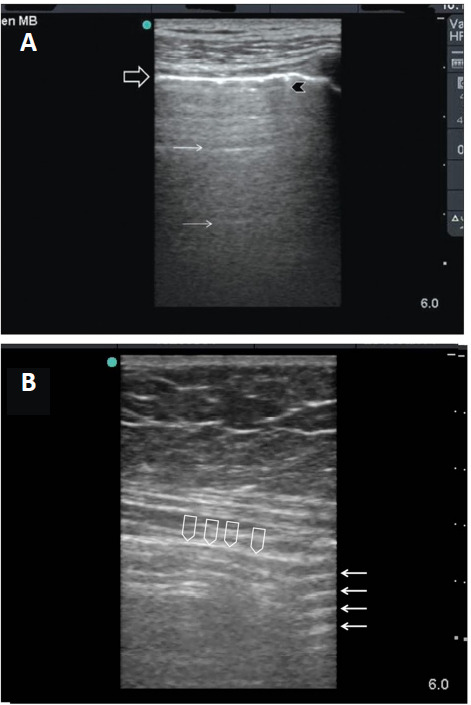
Reverberation artifact of the lung and free intraperitoneal air. (A) Reverberation artifact of the lung occurs as ultrasound waves bounce between the transducer and the pleura. The pleura is shown as a hyperdense white line (black arrow). The reverberation lines (white arrows) represent the repetition of the pleural line. A comet tail artifact is shown (black arrowhead). Reprinted with permission from Abu-Zidan et al.^5^ (B) Sonographic findings of free intraperitoneal air. An enhanced peritoneal stripe sign (arrowheads) does not move with respiration. The reverberation artifact consists of hyperechogenic parallel lines with equal distance between them (arrows). Reprinted with permission from Khan et al.^8^

**The reverberation artifact:** Understanding the biophysics of reverberation artifact, which is the normal sonographic image of air, has a very useful diagnostic value for the presence of pneumothorax or free intraperitoneal air. **Figure 1B** illustrates how reverberation artifact occurs. Both the surface of the ultrasound probe and air have high acoustic impedance. The ultrasound waves that are produced by the probe bounce between these two interfaces while they are gradually being processed by the ultrasound probe and machine over time. For a theoretical example (**Figure 1B**), assume an ultrasound probe emits 100 waves. If all waves reflect back to the probe, and 70 are initially processed while 30 bounce back, then 20 are absorbed and 10 bounce back again. Finally, 7 are received, while 3 bounce back once more. The machine will recognize these waves depending on the time it processes them. Accordingly, it will recognize them as four separate structures with equal distance in depth between them. Since this is a very fast process, the human eye will not be able to observe it. The machine will show an image with horizontal parallel white lines having gradually decreased density (as the number of processed waves gradually decreases), and equal distances between them that equal the distance between the probe and the pleura. These are called A-lines (**Figure 2A**).^5^ A-lines are located deep to the pleural surface preventing the visualization of the deeper structures.

**Free intraperitoneal air:** The same process of knowledge transfer and application of ultrasound from a horse to man has occurred later between the lung and abdomen of human patients when the sonographic findings of pneumothorax have been transferred from the chest to the abdomen. This was based on a similar physical rule, which is “free air inside a body cavity tends to rise to the highest point of that cavity.”^6^

Abdominal gas can be present inside a peristaltic bowel, which is separated from the abdominal fascia by the wall of the bowel. The presence of intraperitoneal air outside the bowel lumen is abnormal, which can be located directly under the abdominal fascia shown by ultrasound as an enhanced peritoneal stripe with reverberation artifact behind it.^7^ When the visceral peritoneum moves over the parietal peritoneum in a normal patient, an experienced operator can observe a fine flickering sliding of the peritoneum. In the presence of free intraperitoneal air, the visceral and parietal peritoneum structures are separated by this air, and the flickering sliding is abolished.

Based on this, three basic sonographic findings have been transferred from the chest to the abdomen, which are: (1) the enhanced pleural line with sliding lung, (2) the reverberation artifact, and (3) the lung point sign. These have the same biophysical principles as (1) the strong enhanced linear hyperechogenic peritoneal stripe associated with the disappearance of sliding of the peritoneum, (2) the reverberation artifact, and (3) the gut point. The “gut point,” which is a diagnostic sign, is the transition point where peritoneal sliding without reverberation artifact, and absence of peritoneal sliding with reverberation artifact can be seen in the same ultrasound image.^7^

These are some of the tips and tricks learned over time that can improve the detection of free intraperitoneal air.^6^,^7^

First: Use the linear-array high-frequency transducer (10–12 MHz). It has less penetration and better resolution compared with the curvilinear transducer (2–5 MHz). It is better for the detection of air that moves up to be superficial.

Second: Start first scanning the abdominal midline in the supine position and then the right upper quadrant between the anterior abdominal wall and the liver (**Figure 2B**).^8^

Third: Compress the caudal end of a parasagittal linear probe firmly and slowly on the abdominal wall over the peritoneal stripe and reverberation artifact. You can observe it being dispersed away from the probe. Remove your pressure slowly and observe how it returns. This is called the” scissors maneuver.”

Fourth: Instruct the patient to breathe deeply and slowly. The enhanced peritoneal stripe will not change while air within the bowl and the intraperitoneal fat will move with inspiration.

Fifth: Turn the patient to be in the left lateral decubitus position. Air will move when changing the patients’ position to be superficial and can obscure the liver.

Sixth: Repeat negative ultrasound examinations in cases of high clinical suspicion of bowel perforation because the diagnosis may become more evident when more intraperitoneal free air accumulates over time.

Initial reports on the use of ultrasound to detect the free intraperitoneal air were very encouraging, having a sensitivity of 94%, a specificity of 64%, a positive predictive value of 97%, and an overall accuracy of 90%.^6^ Nevertheless, recent studies have contradictory results. Sanghvi et al.^9^ prospectively studied the accuracy of POCUS video clips for the diagnosis of pneumoperitoneum by three trained emergency physicians, based on the gas volume. The results were not encouraging. The overall sensitivity was 66%, specificity was 85%, and the area under the curve was only 75%. This study had major limitations affecting its validity. These include: (1) Very high prevalence of pathology in the studied clips (75% of 247 videos), which does not reflect the real incidence in clinical practice. This gave a high false positive predictive value (93%) and a low negative predictive value (45%), (2) The sonographic evaluation was done on the video clips and not on real patients, (3) The sample size of the study was very small compared with the targeted size (only 31 out of 100), (4) The studied patients had elective laparoscopic surgery, not a perforated bowel. In contrast, the results of Herbst et al.^10^ were more encouraging. They studied the accuracy of POCUS video clips in diagnosing small amounts of air (up to 10 mL) injected into 15 fresh human cadavers (3 injections in each) by two trained emergency physicians. Eight were injected into the left upper quadrant while seven were injected into the midline. The sensitivity of POCUS was less in those injected in the left upper quadrant (28%) compared with those injected in the midline (86%). POCUS has been useful in detecting free intraperitoneal air in our own hands. This can be subjective depending on the operator’s experience. This highlights the need to perform large prospective multicentric cohort studies to exactly define the role and generalizability of POCUS in diagnosing free intraperitoneal air.

**Limitations of POCUS:** Despite the fast development in the technology of diagnostic sonography including excellent portable machines and using artificial intelligence there are still barriers that limit the accuracy of ultrasound. It is important to stress that the results of ultrasound studies depend on three factors: (1) the ultrasound machine, (2) the patient, and (3) the operator. The operator’s experience impacts the accuracy of the results. Familiarity with the ultrasound machine, its proper control, the fine movement of the operator, hand eye coordination, and knowledge of radiological anatomy and basic ultrasound biophysics will impact the outcome.

Even when using a well-developed machine, patients’ barriers like very thick fat or subcutaneous emphysema make the ultrasound study more difficult.

**Future developments:** There have been major recent developments in ultrasound technology in the last few years. Portable low-cost lightweight handheld POCUS machines became more affordable to emergency physicians and medical students. This would facilitate the expansion of POCUS training in the undergraduate medical curriculum and its use as a clinical decision tool in clinical practice especially in developing countries. Nevertheless, there are patient safety and security concerns that must be addressed before adopting this strategy. Careful considerations should be taken regarding the efficiency of POCUS training, the credentialling process for POCUS use, regulations of technology approval, and clinical integration of POCUS examinations by properly documenting it in the electronic medical records.^11^

Artificial intelligence is being increasingly used in POCUS, mainly in cardiology, accurately predicting cardiac output and inferior vena cave collapsibility. It could help novice healthcare providers by simplifying the POCUS examination while using hand-held portable or laptop-based POCUS machines. Accordingly, POCUS will lead to proper triage, early diagnosis, and less time stay in the emergency setting, while improving patients’ collaboration, management, and satisfaction. There are still multiple challenges that must be addressed before routinely adopting artificial intelligence including building adequate technical infrastructure, developing legislative guidelines, and protecting patients’ privacy.^12^

There is an obvious need for translational medicine that involves both fundamental research that increases our understanding of this important area and evidence-based research through prospective clinical trials that explore the accuracy and utility of small handheld POCUS machines and artificial intelligence in clinical practice.

**Summary:** We have shown in this perspective that gas ultrasound artifacts have very useful clinical applications if properly understood and utilized. Furthermore, this perspective has highlighted the dynamic transferability of knowledge in biophysics. It showed how a report on reverberation artifact in a horse in 1981 was transferred to human lungs after approximately two decades and then currently to the human abdomen, which has revolutionized its application in intensive care units, emergency medicine, and acute care surgery. No doubt understanding reverberation artifacts and their applications has saved many lives. Nevertheless, the way forward is still challenging including the use of reliable trusted advanced technology such as small portable handheld POCUS machines, proper POCUS training for future doctors, and carefully planned application of artificial intelligence in clinical practice while protecting patients’ safety and privacy.

*The authors thank Miss Mariam Al Ahbabi, Unit Head, Instruction and Scholarly Communication, National Medical Library, United Arab Emirates University for her active role in locating and providing the needed historical papers.*
